# The Effect of Oleuropein on the Treatment of Allergic Rhinitis

**DOI:** 10.1055/s-0045-1809644

**Published:** 2025-08-20

**Authors:** Özge Çaglar Cil, Basak Büyük, Hüseyin Avni Eroglu, Hilal Sehitoglu

**Affiliations:** 1 Department of Otorhinolaryngology, School of Medicine, Çanakkale Onsekiz Mart University, Çanakkale, Turkey; 2 Department of Histology and Embryology, School of Medicine, İzmir Democracy University, İzmir, Turkey; 3 Department of Physiology, School of Medicine, Çanakkale Onsekiz Mart University, Çanakkale, Turkey; 4 Department of Biochemistry, School of Medicine, Çanakkale Onsekiz Mart University, Çanakkale, Turkey

**Keywords:** allergic rhinitis, oleuropein, steroid, treatment, ovalbumin

## Abstract

**Introduction:**

Allergic rhinitis is a disease that negatively affects social and work life and sometimes does not respond to many treatments. Therefore, new treatments are needed. For the prevention and treatment of allergies, oleuropein has been emphasized, and positive results have been shown in the literature.

**Objective:**

To investigate the effects of general and topical oleuropein during the allergic rhinitis period and the histopathological changes in the nasal mucosa compared with steroid nasal sprays and control group in rats.

**Methods:**

We developed an experimental animal model with 44 rats divided into 6 groups. Except for the control group, the allergic rhinitis in the other five groups was created with ovalbumin. As treatment, saline was administered to group 3, steroids, to group 4, oleuropein, to group 5, and steroids and oleuropein were administered to group 6. The effects of the drugs were examined histopathologically and the levels of immunoglobulin E (IgE) in the blood were compared.

**Results:**

Considering the symptomatic findings in rats, we could observe that allergic rhinitis occurred. Based on the IgE levels and histopathological findings, we have statistically shown that oleuropein may be effective in treatment of allergy rhinitis.

**Conclusion:**

Oleuropein has been shown to be useful in the treatment of allergic rhinitis in an animal model, but further studies are needed before it is introduced into the medical practice.

## Introduction

Allergic rhinitis, which affects at least 30% of the world's population, is characterized by inflammation of the nasal mucosa, manifested as a type-1 hypersensitivity reaction. 1 It is a disease that has recently been difficult to treat due to excessive exposure to allergens and increased hyperimmunity. 2

Many medical agents are used in the treatment of allergic rhinitis, such as antihistamines, steroids, montelukast sodium, and immunotherapy. 3 Despite the use of such a variety of drugs, some cases are not fully responsive to the treatments. Therefore, new agents are being studied, and animal studies related to them are also being carried out. 4

Various species in the olive family (which is native to the Mediterranean region and botanically known as *Olea europaea* ) can be used as commercial products, such as food, timber, cosmetics, and medicines. Oleuropein is primarily derived from olives and olive-related products, and it is found in the leaves, fruit (olives), and trunk of olive trees. It is an ester of [2-(3,4-dihydroxyphenyl)-ethanol] (hydroxytyrosol) that has an oleocidic skeleton common to the secoiridoid glucosides of plants in the Oleaceae family. 5 In recent studies, the effects of oleuropein on the prevention and treatment of mast cell-mediated diseases have been emphasized, and several studies have reported that it may be beneficial.

The aims of the present study are to develop an allergic rhinitis model in rats in accordance with the literature, to divide the rats with allergic rhinitis into groups and administer oleuropein or standard therapy to each group, to take tissue samples and compare them histopathologically and immunohistochemically, as well as to compare the levels of immunoglobulin E (IgE) and ovalbumin-specific IgE in the blood of the rats.

## Methods

The present study was carried out in an experimental research center with 44 Wistar Albino rats, 10 weeks old, weighing between 200 and 300 g. The groups were divided as follows: group 1–control group (n = 4); group 2–allergic rhinitis (AR) group (the group in which the AR table was created; n = 8); group 4–AR + saline group (n = 8); group 4–AR + steroids group (n = 8); group 5–AR + oleuropein group (n = 8); and group 6–AR + steroids + oleuropein group (n = 8).

Group 1 is the control group, which was not submitted to any treatments. Group 2 received 1 mg of ovalbumin and 10 mg of aluminum hydroxide intraperitoneally at the same time every day for 14 days. In addition, 10µg/µl ovalbumin was administered to each nostril with a micropipette to increase the allergy for 7 days, and a nasal examination was performed after the intraperitoneal and intranasal administrations of ovalbumin. The nasal symptom scores were independently evaluated by 2 blind observers in experimental groups, with symptoms such as sneezing, itching, and discharge graded on a 0-to-3 point scale.

As in group 2, after the AR table was created to group 3, saline was administered intraperitoneally at the same time every day for 14 days. Group 4 was submitted to the same treatment as group 2 and allergic rhinitis was created, followed by the administration of 0.1 mL of mometasone furoate to each nostril using a micropipette for 14 days.

Group 5 was also submitted to the same treatment as group 2, and the AR table was created, followed by the nasal administration of oleuropein at a dose of 20 mg/kg, diluted in 0.1 mL of saline for 14 days, using a micropipette. In addition, 20 mg/kg of oleuropein were administered orally for another 14 days.

Group 6 was also submitted to the same treatment as group 2, and the AR table was created. Then, 0.1 mL of mometasone furoate was administered to each nostril with a micropipette for 14 days. 6 Additionally, 20 mg/kg of oleuropein diluted in 0.1 mL of saline were administered to both nostrils with a micropipette, followed by the oral administration of the same dose of oleuropein for another 14 days

The animals in groups 1 and 2 were anesthetized with ketamine 50 mg/kg and xylazine 15 mg/kg at the end of the 14 days required to create the AR model. Following the intracardiac blood collection, the nasal cavity, nasal septum, paranasal sinus, and inferior concha tissues were removed en bloc 6 7 and fixed in 10% neutral formalin for a histopathological evaluation. In addition to the 14-day period required for the AR model, the animals in groups 3 to 6 were followed up for another 14 days for treatment, after which they underwent the procedure previously described for groups 1 and 2.

### Histopathological Analysis

The tissues were fixed in a 10% neutral buffered formalin immediately. At the end of the 48-hour fixation period, the tissues underwent routine follow-up procedures and were embedded in paraffin blocks. From the paraffin blocks obtained, sections of 4 μm to 5 μm in thickness were taken with the help of a microtome (Leica Biosystems, model RM 2125 RTS) and stained with the routine hematoxylin and eosin (H&E) staining protocol. The stained sections were evaluated under a biological microscope (Olympus Corporation, model CX43). The histopathological examination was performed on inflammatory cell infiltration, which is one of the parameters mentioned in the literature. 6 8 Histopathological grading was performed according to the literature 9 10 as follows: 0–no damage; 1–slight damage; 2–moderate damage; and 3–severe damage.

### Immunohistochemical Analysis

Immunhistochemical (IHC) staining was performed after the 4-μm sections of paraffin blocks were placed on poly-L-lysine coated slides.

Cyclooxygenase-2 (COX-2), matrix metalloproteinase-9 (MMP-9), tumor necrosis factor-alpha (TNF-α) and interleukin-1 beta (IL-1β) primary antibodies were used in IHC staining to show allergic reaction. The antigen-retrieval method was used in IHC staining. First, the sections were kept in an oven at 65° C for 1 hour and then passed through xylene and alcohol series and turned into ethylenediaminetetraacetic acid (EDTA) at a concentration of 10 mm in a microwave oven at 200 watts. Afterwards, the sections were circumscribed with a peroxidase-antiperoxidase (PAP) pen, and phosphate-buffered saline (PBS; pH of 7.4) was used to wash the tissues dropped in 3% hydrogen peroxide and incubated with primary antibody and marked with 3,3-diaminobenzidine (DAB) chromogen. The sections were cross stained with Harris' hematoxylin, closed, and evaluated under the biological microscope. The evaluation and scoring of the sections were performed as indicated in the literature: 11 12 13 0 = no staining; 1 = slight but identifiable staining; 2 = moderate staining; and 3 = severe staining.

### Statistical Analysis

Data analysis was performed using the IBM SPSS Statistics for Windows (IBM Corp.) software, version 20.0. Frequency, percentage, mean, standard deviation, median, minimum, and maximum values were used in the presentation of the descriptive data. The suitability of the variables to normal distribution was examined using the Shapiro-Wilk test according to the number of subjects in the groups. Nonparametric tests were preferred as analysis methods when the sample size and compliance tests with normal distribution were examined.

The Kruskal-Wallis's test (or the Dunn's test when a significant difference was found) was used to compare variables that were not normally distributed between groups. Statistical significance was set at *p* < 0.05.

## Results

### Symptomatic Findings

The major symptoms of allergic rhinitis (sneezing, nasal itching, and nasal discharge) were subjectively evaluated by two blind observers on the 7th and 14th days between 10 am and 12 pm, immediately after the intranasal administration of ovalbumin OVA application, when each rat was placed in a single cage for 10 minutes in groups of 3 after 10 minutes of adaptation.

The scoring is presented in in Table 1 . We accepted that a successful AR model had been created when the total symptom score was of 5 points. 14

**Table 1:** Allergic rhinitis symptom scoring of the rats in the present study

	0	1 point	2 points	3 points
**Nose scratching movement (number/minute)**	No	2	4–6	**> 6**
**Sneeze (number/10 minutes)**	No	1–3	4–9	≥ 10
**Amount of nasal discharge**	No	Secretion in one nostril	Flowing out of one nostril	Bilateral excessive discharge

When the correlations of the results of both blinded researchers were examined, we observed that they were similar, and the scores of all rats except those in the control group on the 14th day were of 5 points. Therefore, a successful AR model was created in all animals except those in the control group. In addition, the groups were evaluated after the treatments: groups 4, 5, and 6 presented improvement, as their symptom scores decreased to < 3 on average after 14 days of treatment.

### Histopathological Findings

In the present study, the histopathological evaluation was performed in terms of inflammatory cell infiltration. The evaluation was graded as mild, moderate and severe, and for this, all of the mucosa in the section prepared from the tissue removed en bloc was evaluated. The results were as follows:

When the groups 1, 2, and 3 were compared, a significant increase in inflammatory cell infiltration was observed in groups 2 and 3 ( *p* = 0.003 and 0.006 respectively).

**Fig. 1:**
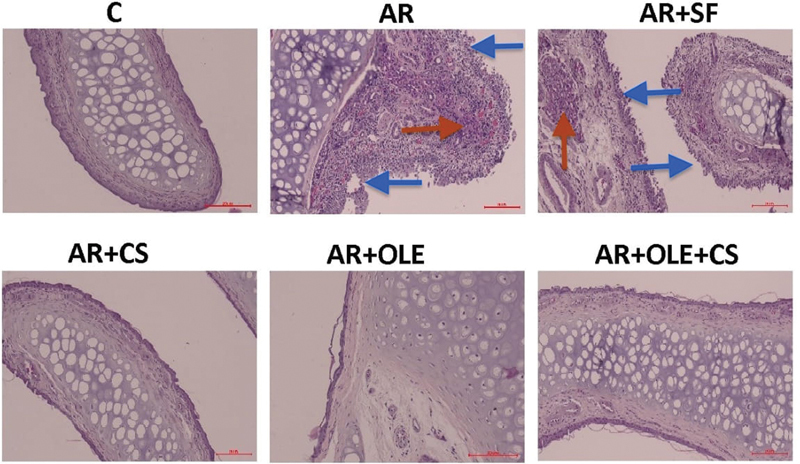
Photomicrography of histological sections of the experimental groups stained with hematoxylin and eosin (H&E). The blue arrows indicate areas of epithelial degeneration, and the red arrows indicate inflammatory cell infiltration (magnification: x200).

When groups 3 and 4 were compared, a significant decrease in inflammatory cell infiltration was observed in group 4 ( *p* = 0.001). When groups 2 and 4 were compared, a significant decrease inflammatory cell infiltration was also observed in group 4 ( *p* < 0.001). In addition, when groups 2, 5, and 6 were compared, a significant decrease in inflammatory cell infiltration was observed in groups 5 and 6 ( *p* = 0.030) ( Table 2 ) ( Fig. 1 ).

**Table 2:** Results of the comparison of inflammatory cell infiltration scores between groups

Group	Inflammatory cell infiltration		*p*	Between groups	Adjusted *p*
	Mean ± standard deviation	Median (minimum–maximum)			
1	–	–	**< 0.001**	1 and 4	1.000
				1 and 6	1.000
				1 and 5	0.616
2	2.8 ± 0.5	3.0 (2.0–3.0)		1 and 3	**0.006**
				1 and 2	**0.003**
				4 and 6	1.000
3	2.6 ± 0.5	3.0 (2.0–3.0)		4 and 5	0.593
				3 and 4	**0.001**
				2 and 4	**0.001**
4	0.3 ± 0.5	0.0 (0.0–1.0)		5 and 6	1.000
				3 and 6	0.062
5	1.5 ± 0.5	1.5 (1.0–2.0)		2 and 6	**0.030**
				3 and 5	0.995
6	0.9 ± 1.1	0.5 (0.0–3.0)		2 and 5	**0.030**
				2 and 3	1.000

**Notes:** Adjusted *p* : Dunn's test; *p* : Kruskal-Wallis test. Values in bold are statistically significant.

### Immunohistochemical Findings

The staining for TNF-α, IL-1, MMP-9, and COX-2 was performed and evaluated.

**Fig. 2:**
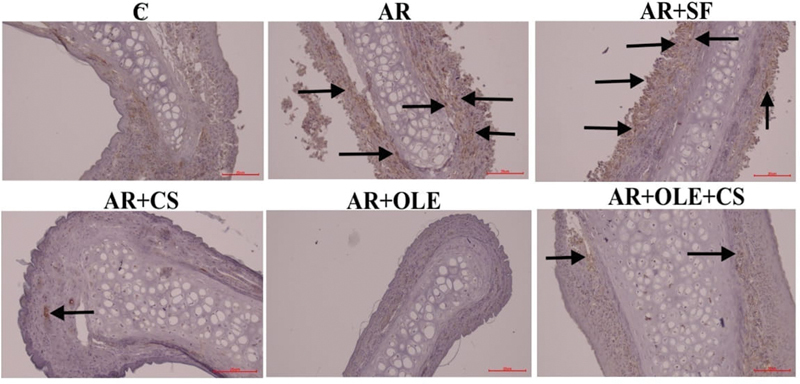
Photographs of immunohistochemical staining of histological sections of the experimental groups regarding cyclooxygenase-2 (COX-2). The arrows indicate immune positive cells (magnification: x200).

Regarding COX-2, when groups 1, 2, and 3 groups were compared, a significant increase was observed in groups 2 and 3 ( *p* = 0.003 and 0.012 respectively). When groups 3 and 4 were compared, a significant decrease was observed in group 4 ( *p* = 0.002). When groups 2 and 4 were compared, a significant decrease was also observed in group 4 ( *p* < 0.001). Moreover, when groups 2, 5, and 6 were compared, a significant decrease was observed in group 5 ( *p* = 0.031) ( Fig. 2 ).

**Fig. 3:**
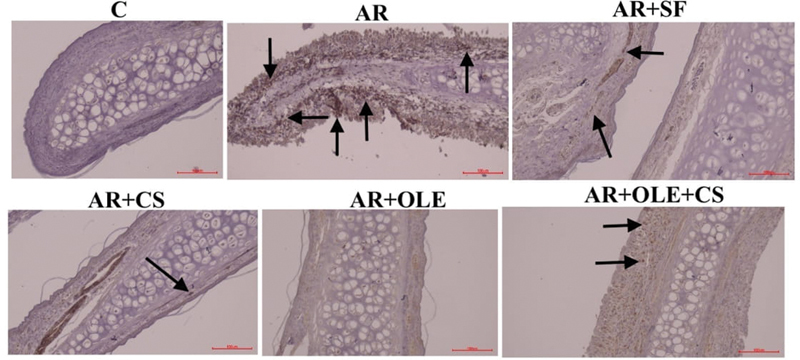
Photographs of immunohistochemical staining of the histological sections of the experimental groups regarding matrix metalloproteinase-9 (MMP-9). The arrows indicate immune positive cells (magnification: x200).

As for MMP-9, when groups 1, 2, and 3 were compared, a significant increase was observed in groups 2 and 3 ( *p* = 0.002 and 0.004 respectively). In the comparison between groups 2 and 4, a significant decrease was observed in group 4 ( *p* = 0.031) ( Fig. 3 ).

**Fig. 4:**
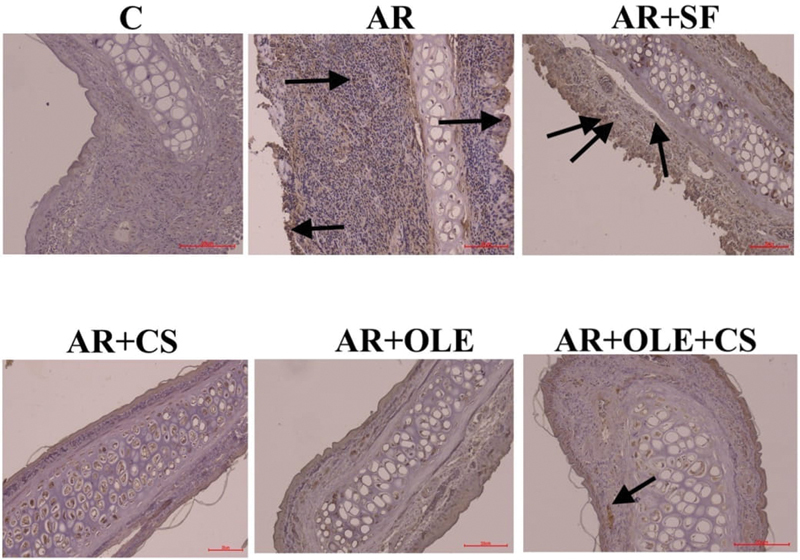
Photographs of immunohistochemical staining of histological sections belonging to the experimental groups regarding tumor necrosis factor-alpha (TNF-α). The arrows indicate immune positive cells (magnification: x200).

Regarding TNF-α, when groups 1, 2, and 3 were compared, a significant increase was observed in groups 2 and 3 ( *p* < 0.001 and *p* = 0.001 respectively). In the comparison of groups 2, 4, 5, and 6, a statistically significant decrease was observed in groups 4, 5, and 6 ( *p* = 0.048, 0.019, and 0.046 respectively) ( Fig. 4 ).

**Fig. 5:**
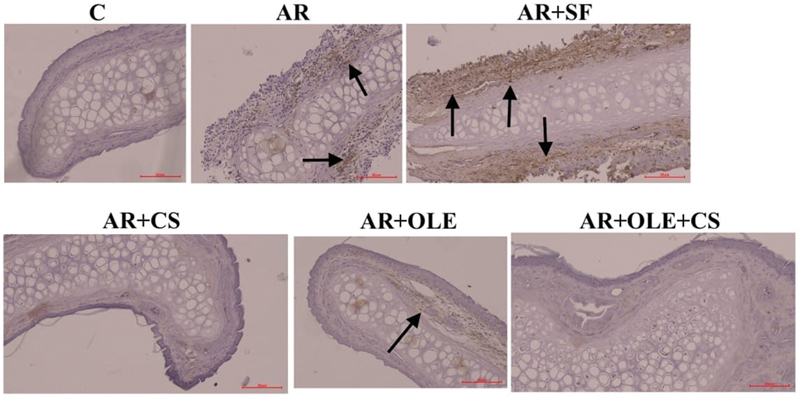
Photographs of immunohistochemical staining of the histological sections of the experimental groups regarding interleukin-1 beta (IL-1β). The arrows indicate immune positive cells (magnification: x200).

As for IL-1β, when groups 4, 5, and 6 were compared with group 1, no significant differences were found regarding groups 4, 5, and 6 ( *p* = 1.000, 0.263, 1.000 respectively). When groups 1, 2, and 3 were compared, a significant increase was observed in groups 2 and 3 ( *p* = 0.006 and 0.001 respectively). In the comparison of groups 3 and 5, a statistically significant decrease was observed in group 5 ( *p* = 0.025) ( Fig. 5 ).

Concerning the IgE and ovalbumin results, in the comparison of groups 2 and 3 with group 1, a statistical difference was found in groups 2 and 3 ( *p* < 0.001 and < 0.01). We also observed a statistical difference between groups 2 and 6 ( *p* < 0.05).

Ovalbumin-specific IgE results were found to be statistically significant in group 2 compared to group 1 ( *p* < 0.001). The ovalbumin-specific IgE levels were also decreased in groups 2 to 6 ( *p* < 0.01).

## Discussion

An important phenolic compound, oleuropein presents antioxidant, antiinflammatory, antiatherogenic, anticancer, antimicrobial, antiviral, hypolipidemic, hypoglycemic, antirheumatic, diuretic, and antipyretic properties, and it also inhibits platelet aggregation. 15 16 17 18

Previous studies have also shown that hydroxytyrosol and oleuropein inhibit mast-cell degranulation: in an experimental study, Şimsek et al., 19 through histopathological and immunohistochemical analyses, showed that oleuropein prevents mast cell-degranulation and conditions such as anaphylactic shock caused by various pharmacological agents.

Throughout the research for a treatment for AR, many animal experiments were conducted to create an AR model through the administration of allergens such as ovalbumin, Japanese cedarwood pollen, Staphylococcal enterotoxin B, *Schistosoma mansoni* egg antigen, toluene 2-4-diisocyanate, and house dust mite allergens. In their study, Wen et al. 20 investigated intranasally administered botulinum toxin A therapy in rats sensitized with ovalbumin.

Similarly, Avincsal et al., 7 in their study on the effect of topical intranasal doxycycline on rats in an AR model, applied a solution with ovalbumin, aluminum hydroxide, and saline intraperitoneally. Guibas et al. 9 used OVA, 10 mg aluminum hydroxide and 1 ml SF solution and applied intraperitoneally. Similarly to the literature, we used ovalbumin in the current study.

Regarding the studies on substances effective against AR in the literature, Lee et al. 21 found that curcumin reduced the IgE-mediated allergic response and allergen-induced mast-cell activity. Sagit et al. 6 investigated the efficacy of quercetin in rats in an experimental AR model, and they found that quercetin is effective against AR.

In one study, 22 oleuropein was shown to inhibit pulmonary inflammation leading to asthmatic fibrosis and alveolar emphysema due to the influx of inflammatory cells in the airways. Therefore, the authors 22 stated that oleuropein could be a promising antiinflammatory agent in the treatment of asthma and chronic obstructive pulmonary disease. In another study, 23 it is once more suggested that olive phenols, particularly hydroxytyrosol and oleuropein, may provide insight into the development of useful tools for the prevention and treatment of mast cell-mediated disorders.

Recent studies 24 on oleuropein have focused especially on its antiallergic and antiinflammatory effects. In the present study, we showed statistically and histopathologically that oleuropein reduces the level of certain inflammatory markers that cause AR, and it also improves allergy symptoms in rats.

## Conclusion

In the current study, we found that oleuropein can be used as an alternative drug in the treatment of AR. However, in order for it to be used routinely in the medical practice, more comprehensive and advanced clinical studies are needed.
